# Reduction in podocyte density as a pathologic feature in early diabetic nephropathy in rodents: Prevention by lipoic acid treatment

**DOI:** 10.1186/1471-2369-7-6

**Published:** 2006-03-15

**Authors:** Brian Siu, Jharna Saha, William E Smoyer, Kelli A Sullivan, Frank C Brosius

**Affiliations:** 1 Departments of Internal Medicine and Physiology, University of Michigan, 1560 MSRB2, 1150 W. Medical Center Dr., Ann Arbor, MI 48109-0676, USA; 2 Department of Pediatrics, University of Michigan, 8220E MSRB III, Ann Arbor, MI 48109-0646, USA; 3 Department of Neurology, University of Michigan, 1311 E Ann, Ann Arbor, MI 48109-0580, USA

## Abstract

**Background:**

A reduction in the number of podocytes and podocyte density has been documented in the kidneys of patients with diabetes mellitus. Additional studies have shown that podocyte injury and loss occurs in both diabetic animals and humans. However, most studies in animals have examined relatively long-term changes in podocyte number and density and have not examined effects early after initiation of diabetes. We hypothesized that streptozotocin diabetes in rats and mice would result in an early reduction in podocyte density and that this reduction would be prevented by antioxidants.

**Methods:**

The number of podocytes per glomerular section and the podocyte density in glomeruli from rats and mice with streptozotocin (STZ)-diabetes mellitus was determined at several time points based on detection of the glomerular podocyte specific antigens, WT-1 and GLEPP1. The effect of insulin administration or treatment with the antioxidant, α-lipoic acid, on podocyte number was assessed.

**Results:**

Experimental diabetes resulted in a rapid decline in apparent podocyte number and podocyte density. A significant reduction in podocytes/glomerular cross-section was found in STZ diabetes in rats at 2 weeks (14%), 6 weeks (18%) and 8 weeks (34%) following STZ injection. Similar declines in apparent podocyte number were found in STZ diabetes in C57BL/6 mice at 2 weeks, but not at 3 days after injection. Treatment with α-lipoic acid substantially prevented podocyte loss in diabetic rats but treatment with insulin had only a modest effect.

**Conclusion:**

STZ diabetes results in reduction in apparent podocyte number and in podocyte density within 2 weeks after onset of hyperglycemia. Prevention of these effects with antioxidant therapy suggests that this early reduction in podocyte density is due in part to increased levels of reactive oxygen species as well as hyperglycemia.

## Background

The clinical hallmarks of diabetic nephropathy include progressive albuminuria followed by a gradual decline in renal function concluding, after 5–15 years, with end-stage renal disease. Glomerular basement thickening and mesangial expansion, due to accumulation of extracellular matrix proteins, have been identified as pathological precursors of these clinical changes [1]. In the last few years, several reports have suggested that loss of glomerular epithelial cells, or podocytes, precedes and predicts the onset of clinical nephropathy and may be an early pathological manifestation of diabetic nephropathy [2-4]. Moreover, it has been postulated that the loss of podocytes could be pathogenically important in leading to glomerular scarring and albuminuria [5].

Meyer and colleagues first showed that podocyte number was reduced in Type 2 diabetic Pima Indian patients. They found that podocytes were decreased in those subjects with microalbuminuria and even more profoundly decreased in those with overt DN [2]. In a prospective follow-up analysis the same authors demonstrated that original podocyte number predicted the rate of increase in albuminuria; those patients with the smallest number of podocytes per glomerulus developed greater increases in albuminuria over a four year period [3]. In a multi-institutional cross-sectional study, Steffes et al. also noted that podocyte number was decreased in patients with Type 1 diabetes [6]. In addition, a report on patients with Type 1 diabetes also showed a decline in podocyte number as nephropathy progressed. However, the number of podocytes was not significantly decreased early in the development of nephropathy nor did it clearly predict the progression of albuminuria. Podocyte loss correlated with increasing albuminuria at follow-up, suggesting that both processes occurred concomitantly [7]. Thus, it remains unclear whether podocyte loss actually precedes or accompanies other early changes of diabetic nephropathy. Moreover, it is uncertain whether podocyte loss actually promotes progression of the disease or is merely a marker thereof.

Studies of podocyte loss in early experimental diabetic animals would provide an avenue for addressing these questions. Therefore, we assessed apparent podocyte number and density in diabetic rats and mice and at early timepoints. Moreover, we determined whether early treatment with insulin or with the antioxidant, α-lipoic acid, resulted in preservation of podocyte density in animals with early diabetes.

## Methods

### Experimental models

The procedures used in this study are in accordance with the guidelines of the University of Michigan Committee on the Use and Care of Animals. Veterinary care was provided by the University of Michigan Unit for Laboratory Animal Medicine. The University of Michigan is accredited by the American Association of laboratory Animal Care. The animal care and use program conformed to the standards in "The Guide for the Care and Use of laboratory Animals," Department of Health, Education, and Welfare Publication No. (NIH) 86-23.

Barrier-sustained Cesarean-delivered male Wistar rats (Harlan, Indianapolis, IN) weighing 200 to 300 g were used. Animals were allowed to acclimatize to their environment for a week before injection. All animals were fasted overnight. Rats in the diabetic group were injected with 50 mg/kg streptozotocin (STZ; Sigma Chemical Co., St. Louis, MO) intraperitoneally while control rats received buffer only. STZ was freshly prepared in 10 mM citrate buffer, pH 5.5 and used within 10 minutes. Rats receiving STZ were given 10% sucrose water in the first 48 hrs after injection to prevent hypoglycemia. Diabetes was confirmed by tail blood glucose levels. Only rats with fasting blood sugar concentrations greater than 300 mg/dl were utilized for the studies. Four separate studies were performed. In the first trial, the effect of STZ diabetes on podocyte number was determined. In experiment two and three, the effect of insulin administration on podocyte number was assessed. In these trials, NPH insulin (1.3 units kg^-1 ^day^-1^) (Eli Lilly and Company, Indianapolis, IN) was injected subcutaneously twice daily (7 AM and 7 PM) beginning three days after STZ treatment and continued for either 2 or 6 weeks. In trial four, the effect of α-lipoic acid on podocyte loss was assessed. In this trial, 100 mg kg^-1^day^-1 ^DL-α-lipoic acid (Sigma) was administered intraperitoneally to a group of STZ diabetic rats starting 48 hours after STZ injection.

For the mouse diabetes experiments, male 6 week-old C57BL/6J mice (Jackson Laboratory, Bar Harbor, Maine) were used. Mice were allowed to acclimate to their environment for 3 days before injection. Mice were fasted for 4 hours and then injected intraperitoneally with 50 mg/kg STZ daily for five consecutive days. Timing of endpoints for the mouse experiments started the day after the final STZ injection.

For both mouse and rat experiments, glucose was measured from a drop of whole blood obtained via the tail vein (HemoCue Glucose Analyzer, Mission Viejo, CA).

### Immunohistochemical analyses

With the rat or mouse under general anesthesia, kidneys were perfused with freshly made PLP fixative (2% paraformaldehyde, 37.5 mM sodium phosphate, pH 7.4, 60 mM L-lysine and 100 mM sodium m-periodate) under constant 100 mmHg pressure via cardiac puncture or through the abdominal aorta after washout with phosphate buffered saline. Kidney cortical regions were dissected and placed into PLP fixative. For the lipoic acid trial, kidney cortical sections were snap-frozen in liquid nitrogen and stored at -70°C prior to fixation.

Sections (3.9 μm-thick) were cut from the PLP-fixed, paraffin embedded kidney samples. These sections were deparaffinized and rehydrated through ethanols (70, 95, 100%). Cryosections (4 μm) from the kidneys obtained from the lipoic acid trial were brought to room temperature and air dried. They were then fixed with pre-chilled (-20°C) acetone for 5 minutes, on ice, and then washed twice with PBS. All sections were incubated in Retrieve One buffer (Signet Laboratories, Inc., Dedham, Massachusetts) at 90°C for two hours to enhance antigen retrieval. Using an ABC Staining Kit (Vector Laboratory Inc., Dedham, MA), podocyte nuclei were detected with a rabbit polyclonal or a mouse monoclonal anti-WT1 antibody (SantaCruz Biotechnology, Inc., Santa Cruz, CA) at a concentration of 2 μg/ml. One set of slides was stained with an antibody to GLEPP1, a podocyte plasma membrane protein, at a concentration of 1/200. The slides for GLEPP1 detection were counterstained with PAS (Periodic acid- Schiff reagent).

### Morphometric analysis

Images from at least 12 sequential glomerular 3.9 μm thick cross sections at approximately the glomerular equator were collected for each histologic section using the MetaMorph Image System (Universal Imaging Corporation; Downingtown, PA) by a blinded observer, as previously reported [5,10]. The mean area of each glomerular profile was measured by manually tracing the glomerular outline on a video screen or encircling the area of interest and calculating that area by computerized morphometry using the Image Measurement System (Jandel Sigma Scan 3.0, USA) or MetaMorph 4.69. The numbers of podocytes per glomerular section and glomerular volume/podocyte (μm^3^) as defined by WT1 or GLEPP1 immunoperoxidase staining was determined. The latter number was calculated as determined in the morphometric analysis of podocyte counting methods by Sanden et al [10]. We utilized identical methods and equipment and utilized the uncorrected glomerular volume/podocyte (Column H, table 1 in reference 10) as reported in that study. GV/P is a variable that incorporates the relationship between both podocyte number and GBM surface area and therefore should be a useful measure of the degree of podocyte reserve [10] and is the reciprocal of podocyte density.

**Table 1:** STZ Rat Characteristics

	Blood glucose concentrations (mg/dl)						Body weight (g)	
		Day 0	Day 3	Day 7	Day 14	End	Day 3	End

Trial 1 (8 wks)	Control		78 ± 5			68 ± 5	270 ± 7	426 ± 18
	STZ		357 ± 24			365 ± 12	237 ± 5	297 ± 18

Trial 2 (2 wks)	Control	53 ± 3	75 ± 5	76 ± 4	62 ± 4		302 ± 3	359 ± 5
	STZ	49 ± 1	363 ± 17	357 ± 24	297 ± 20		274 ± 7	299 ± 9
	STZ + Ins	49 ± 2	147 ± 46	104 ± 38	43 ± 1		290 ± 6	356 ± 7

Trial 3 (6 wks)	Control	51 ± 1	74 ± 4	77 ± 3	65 ± 4	68 ± 3	314 ± 4	467 ± 12
	STZ	48 ± 1	348 ± 23	315 ± 43	305 ± 32	300 ± 6	276 ± 4	347 ± 18
	STZ + Ins	48 ± 2	214 ± 35	60 ± 21	47 ± 3	69 ± 16	278 ± 5	450 ± 6

Trial 4 (6 wks)	Control		N/A			65 ± 7	N/A	455 ± 27
	STZ		377 ± 10			340 ± 60	318 ± 3	327 ± 51
	STZ + LA		369 ± 13			322 ± 44	304 ± 4	306 ± 32

### Data analysis

Data are presented as means ± standard error of the mean. When comparing 2 groups, a Student's t-test was used. For multiple comparisons, an ANOVA followed by Scheffe's post-hoc analysis was performed. P values ≤ 0.05 were considered statistically significant.

## Results

STZ diabetes was induced conventionally in male rats. The rats developed sustained hyperglycemia compared to control rats (Table 1). Subsets of diabetic rats received twice daily insulin for 2, 6 or 8 weeks. Fasting blood glucose levels in insulin-treated rats were significantly lower than untreated STZ-diabetic rats but were still elevated above control values until 2 weeks after initiation of insulin administration. All rats gained weight during the study, although the untreated diabetic rats were statistically lighter than both the control and insulin-treated animals by the end of 2 and 6 weeks (Table 1).

**Figure 1:**
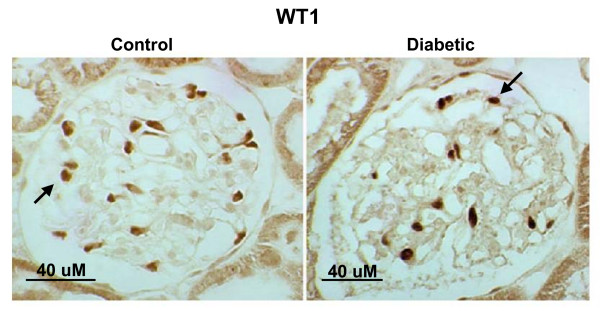
Immunohistological detection of WT1 in control and STZ-diabetic rat glomeruli. Podocytes were detected with antibodies to WT1. WT1 staining is indicated by intense immunoperoxidase activity in podocyte nuclei (arrows).

**Figure 2:**
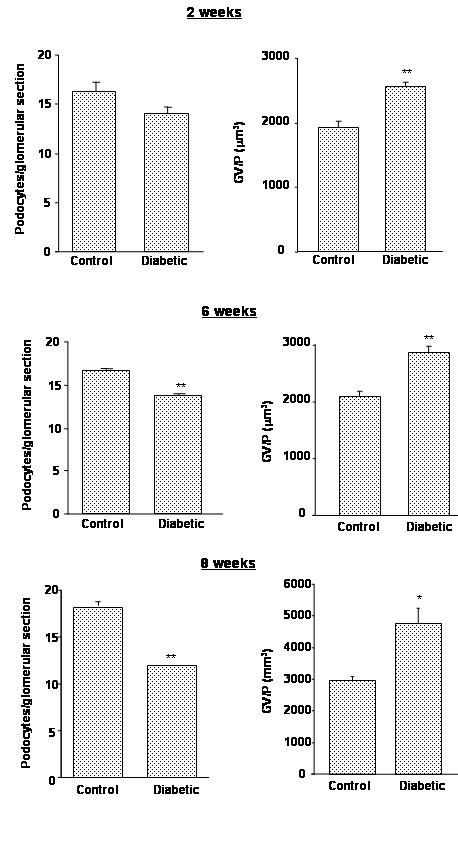
Podocyte number at 2, 6 and 8 weeks after STZ injection in rats. Data are presented as numbers of podocytes per glomerular section and of glomerular volume per podocyte (GV/P [μm^3^]). * = p < 0.01, and ** = p < 0.001 vs. control animals. N = 6 animals per group.

Rats with diabetes showed a reduction in podocyte number as determined by staining for WT1 and GLEPP1 (Fig 1). Podocyte number in STZ diabetes was assessed at 3 time points, 2, 6 and 8 weeks following STZ injection (Fig. 2). Apparent podocyte number, expressed as podocytes/glomerular section was decreased and glomerular volume/podocyte (GV/P) was concomitantly increased in the diabetic rats as early as 2 weeks after STZ injection. When compared to control rats, there was a 14% decline in podocyte number/glomerular section and a 33% reciprocal increase in glomerular volume/podocyte (GV/P) after 2 weeks of diabetes (Fig 2). Similar though somewhat more profound podocyte loss and increase in GV/P were detected in diabetic rats 6 and 8 weeks after STZ injection (Fig 2).

**Figure 3:**
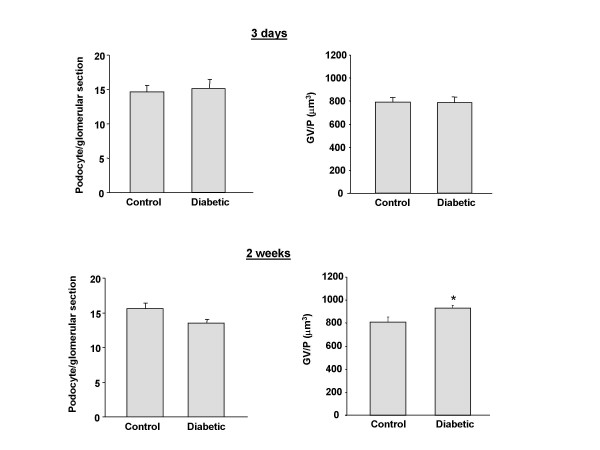
Podocyte number at 3 days and 2 weeks after end of the 5 day STZ injection protocol in mice. Data are presented as numbers of podocytes per glomerular section and of glomerular volume pre podocyte (GV/P [μm^3^]). * = p < 0.05 vs. control animals. N = 5 animals per group.

Male C57BL/6 mice were made diabetic with repeated low dose STZ injection. Their weights and glucose concentrations are displayed in Table 2. As in the rat model, these diabetic mice showed a decline in podocyte number 2 weeks after the final STZ injection (Fig. 3). There was a 15% decline in the number of podocytes per glomerular section and a 15% increase in GV/P at 2 weeks after the end of the STZ injection period. Importantly, there was no difference in podocyte numbers in streptozotocin-treated mice 3 days after the end of the 5-day STZ injection protocol (Fig 3). This strongly suggests that STZ does not have a direct toxic effect on podocytes in this model.

**Table 2:** STZ Mouse Characteristics

	Blood glucose (mg/dl)		Body weight (g)	
	3 days	2 weeks	3 days	2 weeks

Control	133.5 ± 5.4	123.2 ± 5.1	25.8 ± 0.3	27.8 ± 0.4
STZ	171.8 ± 10.2	303.4 ± 16.5	23.5 ± 0.4	25.0 ± 0.6

**Figure 4:**
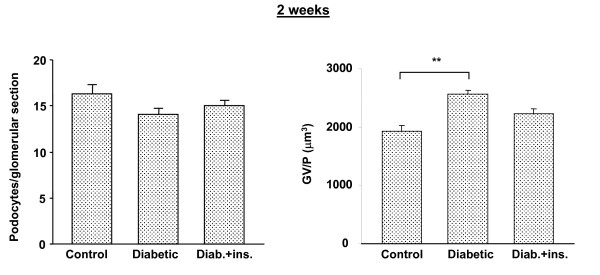
Podocyte number at 2 weeks after STZ injection in rats. Diab. + ins. = data for diabetic rats that received twice daily insulin injections beginning 3 days after STZ injection. Data are presented as numbers of podocytes per glomerular section and of glomerular volume pre podocyte (GV/P [μm^3^]). ** = p < 0.001 vs. control animals. N = 6 animals per group.

**Figure 5:**
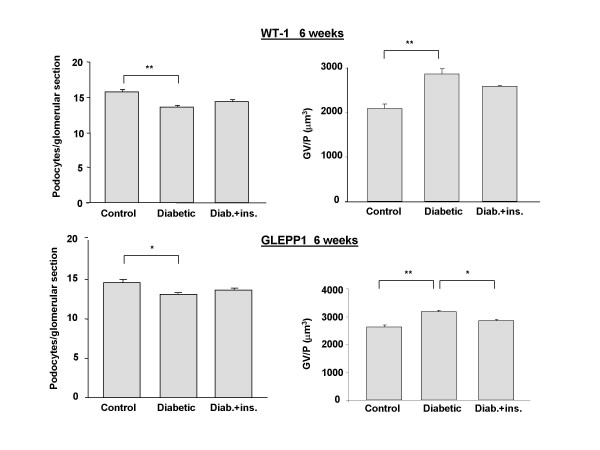
Podocyte number at 6 weeks after STZ injection in rats. Diab. + ins. = data for diabetic rats that received twice daily insulin injections beginning 3 days after STZ injection. Data are presented as numbers of podocytes per glomerular section and of glomerular volume pre podocyte (GV/P [μm^3^]). Six week trial data were combined for this analysis. N = 11 (Control), 10 (Diabetes) or 6 (Diab. + ins). Staining for both WT1 and GLEPP1 is displayed. * = p < 0.05, ** = p < 0.0001

**Figure 6:**
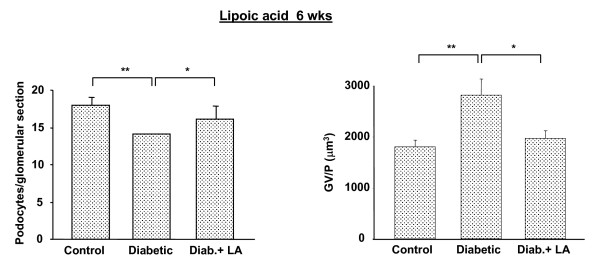
Podocyte number at 6 weeks after STZ injection in rats. Diab. + LA = data for diabetic rats that received daily α-lipoic acid injections beginning 2 days after STZ injection. Data are presented as numbers of podocytes per glomerular section and of glomerular volume pre podocyte (GV/P [μm^3^]). * = p < 0.05; ** = p < 0.001. N = 5 animals per group except in Diabetes group which was 4 animals.

Additionally, at both 2 and 6 weeks post STZ injection, diabetic rats treated with insulin showed an intermediate phenotype between normal and diabetic rats that did not receive insulin treatment (Figs. 4 and 5). Thus, insulin treatment appeared to prevent some of the reduction in podocyte density accompanying STZ diabetes. Daily treatment with α-lipoic acid had a more substantial normalizing effect on podocytes/glomerular section and GV/P. Diabetic rats receiving this agent, while similarly hyperglycemic to diabetic rats that did not receive it, had almost normal numbers of podocytes per glomerular section and GV/P (Fig. 6).

## Discussion

In the current study we have determined that podocyte density and apparent glomerular podocyte number are substantially reduced in rats and mice injected with STZ, a model of Type 1 diabetes mellitus, quite early after initiation of diabetes. Reduction in apparent podocyte  number and podocyte density occurred as early as 2 weeks after STZ  injection, and appeared to worsen somewhat by 6 and even further by 8  weeks after STZ administration. Importantly, STZ did not appear to have an acute toxic effect on podocytes, at least in the murine model, since 3 days after completion of the five day STZ injection protocol apparent podocyte number and density was completely normal in STZ treated mice. Also significantly, insulin treatment had only a modest effect in maintaining podocyte number, despite inducing a substantial improvement in fasting blood glucose levels by 2 weeks after initiation of diabetes. In contrast, administration of the antioxidant, α-lipoic acid was more effective in maintaining podocyte number and density. These latter data suggest that near normalization of glucose levels, even as early as 2 weeks after initiation of STZ-diabetes, cannot fully prevent the reduction in podocyte number, possibly because the early effects of hyperglycemia are dominant in this model. In addition, the protection with α-lipoic acid suggests that oxidant-mediated injury plays a role in the early diminution in podocyte density consistent with other effects of oxidants in mediating or enhancing diabetic complications [e.g., [12]].

Our methods of podocyte counting relied on accurately identifying podocyte nuclei (WT-1) or cytoplasm (GLEPP1). The WT-1 staining tends to be more precise since it is difficult to distinguish the cytoplasmic extent of any single podocyte using these methods. As noted by these investigators, a  potential disadvantage of this method is that podocyte nuclei are  not uniformly WT-1 positive under some pathologic circumstances [10]. As noted by these investigators, a potentially disadvantage of this method is that podocyte nuclei are not uniformly WT-1 positive under some pathologic circumstances ([10]). However, as they also noted, there are no data to indicate that podocytes in DN are WT-1 negative. In addition, WT-1 is an excellent marker of the normal mature differentiated podocyte and even podocytes with effaced foot processes remain WT-1 positive. Hence, if a podocyte is WT-1 negative, then it is likely to be severely and perhaps terminally damaged. Thus, we believe that our data are consistent with true and quite early reductions in podocyte density in experimental diabetic nephropathy and at the least reflect severe podocyte damage.

Although the morphometric methods utilized in this study are somewhat different from those developed by Weibel and Gomez and Cavalieri, they clearly assess very similar parameters and have been well validated in the previous publications [5,10]. Nonetheless, since we did not survey glomerular serial sections in these studies, nor investigate both thick and thin sections of the same glomerulus, we cannot claim that we have demonstrated an absolute reduction in numbers of podocytes/glomerulus. The increase in glomerular size in diabetes may have contributed to the reduction in podocytes/glomerular section. However, it is clear that glomerular density was substantially reduced and the glomerular volume subsumed by each podocyte was increased in these Type 1 diabetic models. Although there appears to be a great deal of precision using these methods, as noted by the small variances in each study, there was a substantially greater reduction in apparent podocyte number after 8 weeks than after 6 weeks of STZ-diabetes (34 vs. 18 %). Whether this represents true continued podocyte loss over the intervening time period is uncertain, and would need to be validated by a larger single trial in which all animals were made diabetic at the same time followed by assessment of podocyte loss in samples at regular intervals.

Previously, several studies in Type 1 and Type 2 diabetic patients have suggested that diabetes leads to a reduction in glomerular podocyte number as a relatively early marker of diabetic nephropathy [2-4,6]. Moreover, Meyer and colleagues showed that the number of podocytes per glomerulus at the start of the study was predictive of the degree of albuminuria after a four year follow-up, suggesting that podocyte loss precedes and predicts progressive diabetic nephropathy [3]. More recently, in a study of Italian patients with Type 2 diabetes mellitus, substantial abnormalities in podocyte structure and density were found to occur during the early stages of diabetic nephropathy [13]. Specifically, glomerular podocyte density declined early in diabetes and correlated inversely with albumin excretion rates, although the number of podocytes per glomerulus did not correlate with progression of disease. These findings prompted the authors to conclude that the density, not the absolute number, of podocytes may be important for the progression of nephropathy in Type 2 patients. In a report by Steffes et al., for the International Diabetic Nephropathy Study Group, Type 1 diabetic patients were found to have a significantly lower number of podocytes than age-matched control subjects [6]. A more recent study has also shown that podocyte loss accompanies Type 1 diabetes but that this podocyte loss did not appear to occur as early as in Type 2 patients [7]. Although it reported that podocyte number was lower in diabetic patients than in nondiabetic controls and that the surface covered by each podocyte increased gradually with time, the absolute number of podocytes did not predict the degree of albuminuria. Nonetheless, in the longitudinal component of the study, there was a significant correlation between the decrease in podocyte number and the degree of albuminuria over the period from the baseline observation to follow-up three years later [7]. From all of these human studies, it remains impossible to conclude whether decreased podocyte number or density actually results in increased albuminuria and progressive nephropathy or whether the podocyte changes and albuminuria are simply associated findings.

Since the human studies cannot ascertain whether the decrease in podocyte number causes or simply correlates with advancing albuminuria and nephropathy, it would be beneficial to identify animal models that recapitulate similar changes. Such models would aid the investigation of the mechanisms by which diabetes results in podocyte loss and allow determination of whether podocyte loss directly results in albuminuria. The early podocyte loss that occurs in humans with diabetic nephropathy appears to occur quite early in both rat and mouse STZ diabetes. While the changes are more profound in the rat models, similar although less substantial reductions in podocyte density occur in mice after only 2 weeks of STZ diabetes. Although the current study is the first to show that experimental murine and rat models of diabetes undergo very early podocyte loss, several previous studies using experimental models of diabetes have demonstrated podocyte loss or abnormalities after longer periods of diabetes. Gross and colleagues have shown that similar reduction in podocytes occurs after 6 months of streptozotocin (STZ) diabetes in rats [14] and that loss of podocytes in this model was prevented by treatment with the angiotensin converting enzyme inhibitor, trandolopril. In a separate study, these investigators also showed substantial podocyte loss after 6 months of diabetes in the SHR/N-cp rat, a model of type II diabetes that spontaneously develops pronounced abnormalities in renal histology. In comparison to STZ-diabetic rats, which develop relatively modest glomerular changes at 6 months of diabetes, glomeruli from the SHR/Ncp rats contained fewer and larger podocytes, smaller mesangial cells and a more expanded mesangial matrix [15].

Mifsud and colleagues did not examine podocyte number but documented that the number of slit pores per unit length of glomerular basement membrane in rat glomeruli was decreased 24 weeks after STZ injection, consistent with podocyte foot process broadening [9]. These changes, as well as the increased albumin excretion seen in this model, were also ameliorated by treatment with either an angiotensin receptor blocker or an angiotensin converting enzyme inhibitor [9]. Gassler and colleagues studied nephron degeneration in Zucker fa/fa male rats, a model of Type 2 diabetes, at 10 months of age and concluded that degeneration began with damage to podocytes [16]. They demonstrated sclerosis in approximately 25% of the diabetic glomeruli and found evidence of more extensive, "pre-sclerotic" podocyte injury including foot process effacement, pseudocyst formation, and accumulation of lysosomal granules and lipid droplets in podocyte cytoplasm. These podocyte changes appeared to play a significant role in the progression of the segmental glomerular injury to global sclerosis as well as to the degeneration of the corresponding tubule. Hoshi and colleagues also found electron microscopic evidence of podocyte degeneration and the development of tuft adhesions that they concluded were responsible for the glomerular sclerosis in the same model [8].

In addition, several studies have found that diabetes was associated with a significant reduction in expression of the podocyte slit diaphragm protein, nephrin, in both human [17] and animal models [18] of diabetes. However, in the studies in STZ diabetes in rats there was no change in nephrin expression after one week of diabetes [17]. Since our data indicate that podocyte changes occur quite early in STZ diabetes and is statistically significant as early as 2 weeks after induction of diabetes, it seems likely that the change in nephrin expression was either a response to podocyte injury or stress, or developed independently of the early podocyte changes found in this model.

The biochemical and metabolic signals that result in diabetic glomerulopathy have been the subject of investigation over the past several decades. A unifying factor in promoting most if not all of the abnormalities found in the diabetic glomerulus appears to be the increase in mitochondrial oxidative stress generated by enhanced glucose metabolic flux [19,20]. For these studies, we used a potent inhibitor of mitochondrial superoxide generation, α-lipoic acid, which virtually eliminated the effects of STZ-diabetes on reduction in podocyte density and apparent number, supporting the notion that mitochondrial reactive oxygen species are critical in these early changes of diabetic glomerulopathy.

Since STZ also has nephrotoxic effects, especially when given at high doses, it is conceivable that STZ had a direct toxic effect on podocytes and that some of the effect of α-lipoic acid was to protect against such toxicity. While our studies do not absolutely rule this out, we feel this is unlikely for several reasons: 1) other models of diabetic nephropathy that arise spontaneously show similar decreases in podocyte number and density [e.g., [15]]; 2) relatively low dose STZ as used in these experiments has not been associated with proximal tubule toxicity and the proximal tubule cell should be especially sensitive to STZ since, like the pancreatic β cell, it expresses high levels of the facilitative glucose transporter, GLUT2 which transports STZ into cells [21], 3) finally and most importantly, we demonstrated that no changes in podocyte density or apparent number occurred within 3 days after completion of a 5-day low dose STZ protocol in mice, which strongly suggests that STZ has no acute podocyte toxicity.

Although the early apparent loss of podocytes in the STZ diabetic rat and mouse models implicates podocyte damage in the pathogenesis of diabetic nephropathy, it is also striking that such changes precede albuminuria and glomerulosclerosis by at least several months. Moreover, the STZ rat and mouse models, as is true for most animal models of diabetic complications, fail to develop classic changes of human diabetic nephropathy. Thus it seems likely that the early podocyte changes, though important, may not be sufficient to lead to progressive diabetic nephropathy and renal failure. It may be that a certain threshold of podocyte damage must be achieved before sclerosis occurs, as suggested by Kim et al. [5]. Conversely, nephrosclerosis may be dependent on mesangial and other factors that are not directly affected by podocyte changes, as suggested by many studies over the past 20 years (for an overview, see reference 22). Nonetheless, the sensitivity of the podocyte to hyperglycemic damage is now evident. These early changes in podocytes may lead to long-term glomerular responses that are critical for the development of diabetic nephropathy.

## Conclusion

Reduction in podocyte density that occurs in humans with early diabetic nephropathy is recapitulated in rat and mouse models of diabetes. Some of this reduction in podocyte density is likely due to podocyte loss, and can be prevented by treatment with a powerful antioxidant, suggesting that podocytes undergo oxidant injury quite early in diabetic kidney disease. It should now be possible to utilize molecular methods in murine and other animal models to further determine the mechanisms of early podocyte damage and the structural and functional sequelae of podocyte loss in diabetic nephropathy.

## Competing interests

The author(s) declare that they have no competing interests.

## Authors' contributions

BS carried out rat diabetes studies, carried out and directed the morphometry analysis and assisted with drafting of the manuscript. JS carried out the podocyte counting and morphometry analysis. WES assisted with the experimental design, data analysis and manuscript preparation. KAS carried out rat diabetes studies and assisted with the morphometry analysis. FCB conceived of the study, and participated in its design and coordination and helped to draft the manuscript. All authors read and approved the final manuscript.

## Pre-publication history

The pre-publication history for this paper can be accessed here:


